# Algorithm based online speech evaluation - the new horizon

**DOI:** 10.1192/j.eurpsy.2022.1937

**Published:** 2022-09-01

**Authors:** E. Vashdi

**Affiliations:** Yaelcenter institute, Clinic, Aloney Aba, Israel

**Keywords:** Apraxia of speech, algorithm, VML method, Treatment

## Abstract

**Introduction:**

The VML method was developed and designed to treat Apraxia of speech focusing on the Autistic population. After experiencing over 2000 children in many countries around the world, we have developed an algorithm which represents the VML analysis process. The algorithm includes almost 1000 conditions and was found reliable with copying the in-person VML evaluation. The algorithm generates a treatment program with 95% accuracy of the elected treatment topics.

**Objectives:**

The objective of the VML software is to enable the VML analysis and treatment at low cost to wide population around the world, at home. The program includes main treatment topics, detailed exercises, picture and videos demonstrating the proposed treatment and general guidelines. The software users are supported by the VML experts around the world.

**Methods:**

Based on the algorithm, we have developed a software which can produce a highly detailed motor speech treatment program. The software is web based, available now in English, Mandarin and soon in other languages as well. The user is required to fill in the speech data using the software interface.

**Results:**

The uniqe sofetware was tested and found to have 90% reliability rate in comparison to a VML expert treatment program. In addition it was found to have the ability to over come mild evaluation mistakes while producing an effective treatment program.

**Conclusions:**

The MYVML evaluation software is innovation in the field of speech treatment, striving to share the knowledge and give the treatment tool to as many practitioners and families as possible.

**Disclosure:**

I am the developer of the VML software described in the abstract

